# Let-7b/c Enhance the Stability of a Tissue-Specific mRNA during Mammalian Organogenesis as Part of a Feedback Loop Involving KSRP

**DOI:** 10.1371/journal.pgen.1002823

**Published:** 2012-07-26

**Authors:** Emanuela Repetto, Paola Briata, Nathalie Kuziner, Brian D. Harfe, Michael T. McManus, Roberto Gherzi, Michael G. Rosenfeld, Michele Trabucchi

**Affiliations:** 1 INSERM U1065, Centre Méditerranéen de Médecine Moléculaire (C3M), Team 10 “Control of Gene Expression,” Nice, France; 2 University of Nice Sophia-Antipolis, Faculty of Medicine, IFR50, Nice, France; 3 Gene Expression Regulation Laboratory, IRCCS San Martino-IST, Genova, Italy; 4 Department of Molecular Genetics and Microbiology, University of Florida, Gainesville, Florida, United States of America; 5 UCSF Diabetes Center, Department of Microbiology and Immunology, University of California San Francisco, San Francisco, California, United States of America; 6 Howard Hughes Medical Institute, Department and School of Medicine, University of California San Diego, La Jolla, California, United States of America; The University of Kansas Medical Center, United States of America

## Abstract

Gene silencing mediated by either microRNAs (miRNAs) or Adenylate/uridylate-rich elements Mediated mRNA Degradation (AMD) is a powerful way to post-transcriptionally modulate gene expression. We and others have reported that the RNA–binding protein KSRP favors the biogenesis of select miRNAs (including let-7 family) and activates AMD promoting the decay of inherently labile mRNAs. Different layers of interplay between miRNA– and AMD–mediated gene silencing have been proposed in cultured cells, but the relationship between the two pathways in living organisms is still elusive. We conditionally deleted *Dicer* in mouse pituitary from embryonic day (E) 9.5 through Cre-mediated recombination. *In situ* hybridization, immunohistochemistry, and quantitative reverse transcriptase–PCR revealed that Dicer is essential for pituitary morphogenesis and correct expression of hormones. Strikingly, αGSU (alpha glycoprotein subunit, common to three pituitary hormones) was absent in *Dicer*-deleted pituitaries. αGSU mRNA is unstable and its half-life increases during pituitary development. A transcriptome-wide analysis of microdissected E12.5 pituitaries revealed a significant increment of KSRP expression in conditional *Dicer*-deleted mice. We found that KSRP directly binds to αGSU mRNA, promoting its rapid decay; and, during pituitary development, αGSU expression displays an inverse temporal relationship to KSRP. Further, let-7b/c downregulated KSRP expression, promoting the degradation of its mRNA by directly binding to the 3′UTR. Therefore, we propose a model in which let-7b/c and KSRP operate within a negative feedback loop. Starting from E12.5, KSRP induces the maturation of let-7b/c that, in turn, post-transcriptionally downregulates the expression of KSRP itself. This event leads to stabilization of αGSU mRNA, which ultimately enhances the steady-state expression levels. We have identified a post-transcriptional regulatory network active during mouse pituitary development in which the expression of the hormone αGSU is increased by let7b/c through downregulation of KSRP. Our study unveils a functional crosstalk between miRNA– and AMD–dependent gene regulation during mammalian organogenesis events.

## Author Summary

Pituitary gland development has served as a powerful model system by providing insights into the molecular strategies that underlie emergence of distinct cell types from a common primordium during organogenesis, permitting the discovery of novel regulatory mechanisms. Adenylate/uridylate–rich elements Mediated Degradation (AMD) is a central post-transcriptional mechanism to control the half-life of labile transcripts that contributes to their temporal and spatial expression regulation during tissue differentiation and development. Here, we report that the targeted deletion of *Dicer* in developing mouse pituitary reveals a role of miRNAs in regulating mRNA stability networks through a direct expression control of the AU-rich element mediated degradation regulator, KSRP. KSRP in turn directly regulates the stability of alpha glycoprotein subunit mRNA, common to three pituitary hormones, ultimately leading to an upregulation of its steady-state level expression. These findings define a hierarchical mechanism by which the upregulation of specific miRNAs controls the expression of KSRP and the subsequence decay rate of its target labile mRNAs during mammalian organogenesis.

## Introduction

MicroRNAs (miRNAs) are small regulatory RNAs that mediate post-transcriptional silencing of specific target messenger RNAs (mRNAs) [1]. miRNAs control a wide range of cellular activities, including development, immune function and neuronal plasticity [1]. The importance of miRNAs is underscored by mounting evidence that misregulation of specific miRNA pathways is associated with complicated health afflictions such as cancer [2]. Mature miRNAs are formed by two sequential processing reactions: primary transcripts of miRNA genes (pri-miRNAs) are first processed into hairpin-containing intermediate precursors (pre-miRNAs) by the Drosha microprocessor complex, and pre-miRNAs are then cleaved into mature miRNAs by Dicer [3]. Finally, mature miRNAs are loaded in the RNA-induced silencing complex (RISC) to mediate degradation and/or block translation of specific target mRNAs via Watson-Crick base pairing partial sequence complementarity [1].

The decay rate of mRNAs varies considerably from one species to another and plays an important role in modulating gene expression [4]. mRNA decay rate is increased by the presence of specific AU-rich sequences called ARE (AU-rich element) within the mRNA molecule. AREs can be present in the 3′UTRs of short-lived transcripts and numerous RNA-binding proteins have been described to bind them (ARE-binding protein (ARE-BP)) [5]. ARE-BPs regulate the recruitment of mRNA decay enzymes to target mRNAs . Some ARE–BPs, such as tristetraprolin (TTP), butyrate response factor 1 and 2 (BRF1 and BRF2), T-cell intracellular antigen 1 (TIA-1), KH-type splicing regulatory protein (KSRP) promote decay of ARE-containing RNAs, while others, such as Hu antigen R (HuR), stabilize them. AU-rich element RNA-binding protein 1 (AUF1) either stabilizes or destabilizes ARE-containing mRNAs depending on the experimental systems used [5]. Different layers of interplay between miRNA- and AMD (Adenylate/uridylate–rich elements Mediated mRNA Degradation)- mediated gene silencing have been proposed in cultured cells but the relationship between the two pathways in living organisms is still elusive [6], [7], [8], [9].

KSRP is a multifunctional RNA binding protein that modulates many steps of RNA life including pre-mRNA splicing, ARE-mediated mRNAs decay, and maturation of select miRNAs from precursors [10]. Studies in cells and animal models revealed that KSRP is essential for the control of cell proliferation and differentiation as well as for the regulation of the innate immune response against viral infections, and the response to DNA damage [11], [12].

In this report we have investigated a post-transcriptional regulatory network active during mouse pituitary development that integrates miRNA-dependent and AMD-mediated gene silencing. Temporal and spatial regulation of gene expression is essential for cell fate determination in pituitary where distinct hormone-producing cell types arise from a common ectodermal primordium [13], [14]. The mature anterior pituitary gland contains distinct hormone-producing cell types, including corticotropes secreting adrenocorticotrophic hormone (ACTH), a proteolytic product of proopiomelanocortin (POMC); thyrotropes secreting thyroid-stimulating hormone (TSH); somatotropes, secreting growth hormone (GH); lactotropes, secreting prolactin (PRL); gonadotropes, secreting luteinizing hormone (LH) and follicle-stimulating hormone (FSH). TSH, LH, and FSH are heterodimeric glycoproteins containing a common α-subunit (αGSU) and a hormone-specific β-subunit (TSHβ, LHβ, and FSHβ).

Here, we demonstrated that Dicer is essential for mouse pituitary morphogenesis and correct expression of hormones with αGSU being absent in *Dicer*-deleted pituitaries. We found a negative feedback regulatory mechanism centered on KSRP that promotes let7b/c maturation from precursors and is, in turn, downregulated by let7b/c themselves. KSRP downregulation proved to be essential for the temporarily and spatially correct expression of αGSU through stabilization of its mRNA. Our studies unveil a functional crosstalk between miRNA- and AMD- dependent gene regulation during cell lineage determination in an animal model.

## Results/Discussion

**Figure 1:**
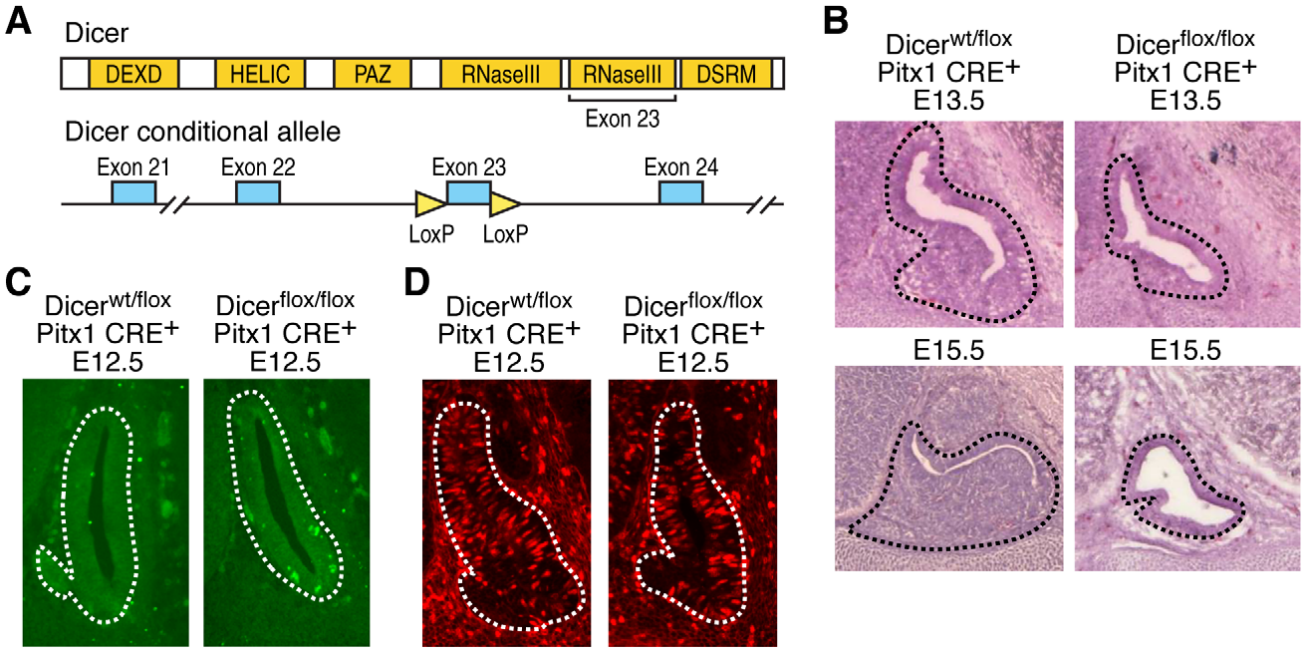
Deletion of Dicer during pituitary development leads to cell death and hypoplastic phenotype. (A) Schematic representation of the *Dicer* conditional targeting construct. (B) Hematoxylin and eosin staining of control versus *Dicer*-deleted pituitaries; representative sagittal sections of E13.5 and 15.5 pituitaries are shown. (C) Apoptosis is observed by TUNEL in control or *Dicer*-deleted pituitaries at E12.5; a representative sagittal section of pituitary gland is shown. (D) BrdU-positive cells in control or *Dicer*-deleted pituitaries at E12.5 by immunohistochemistry; a representative sagittal section of pituitary gland is shown.

**Figure 2:**
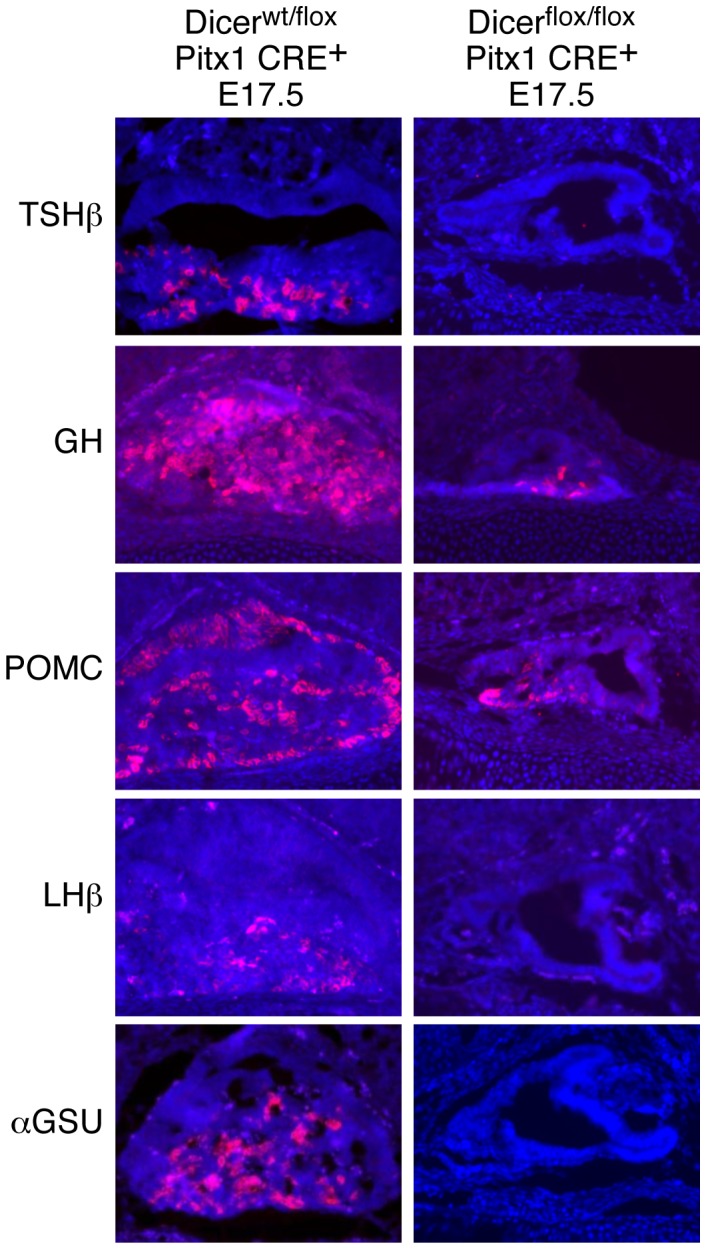
Dicer is required for cell-type-specific pituitary hormone expression. Protein expression of TSHβ, GH, POMC (expression was detected using anti-ACTH immunoglobulin G), LHβ, and αGSU in control or *Dicer*-deleted pituitaries at E17.5 by immunohistochemistry; a representative sagittal section of pituitary gland is shown.

*Dicer*-null mouse embryos die at E7.5 [15], before pituitary development starts. Therefore, in order to investigate miRNAs relevance in modulating pituitary organogenesis, we generated a pituitary-specific *Dicer* gene deletion by crossing *Dicer^flox/flox^* [16] and *Pitx1 CRE^+^* mouse lines (Figure 1A) [17]. *Pitx1 CRE^+^* mice selectively express Cre recombinase in the pituitary primordium and have been shown to execute effective recombination of the *ROSA26* locus in nearly all pituitary cells by E9–E9.5 [17]. Reverse transcription followed by PCR (RT-PCR) of mRNAs from microdissected E12.5 pituitary glands revealed a complete loss of the Dicer second RNaseIII domain in *Dicer^flox/flox^ Pitx1 CRE^+^* (Figure S1A), which results in miRNA processing blockade in comparison with control *Dicer^wt/flox^ Pitx1 CRE^+^* and *Dicer^wt/wt^ Pitx1 CRE^+^*, as examined by Northern blot using a representative miRNA antisense probe (let-7c, Figure S1B). *Dicer^flox/flox^ Pitx1 CRE^+^* mice died shortly after birth due to cleft palate (data not shown). *Dicer*-deleted pituitaries appeared hypoplastic with an enlarged lumen (Figure 1B) probably as a result of cell death (Figure 1C and Figure S1C) indicating that Dicer is required for cell survival during pituitary development. Similarly, an increased apoptosis has been observed in the other conditional *Dicer* knockout mice, such as in limb [16], lung [18], skeletal muscle [19], and brain [20]. Cell proliferation, assessed by BrdU incorporation and Ki-67 expression, was unaffected in mutant mice (Figure 1D and Figure S1D). Importantly, at E17.5, expression of TSHβ, LHβ and αGSU (also known as Cga) was undetectable by immunostaining in *Dicer*-deleted pituitaries (Figure 2). Expression of GH and POMC, markers for somatotrope and the corticotropes/melanotrope cell types, respectively, was also remarkably decreased in *Dicer*-delete pituitaries. Therefore, Dicer is essential for the pituitary morphogenesis and the correct expression of all pituitary hormones.

**Figure 3:**
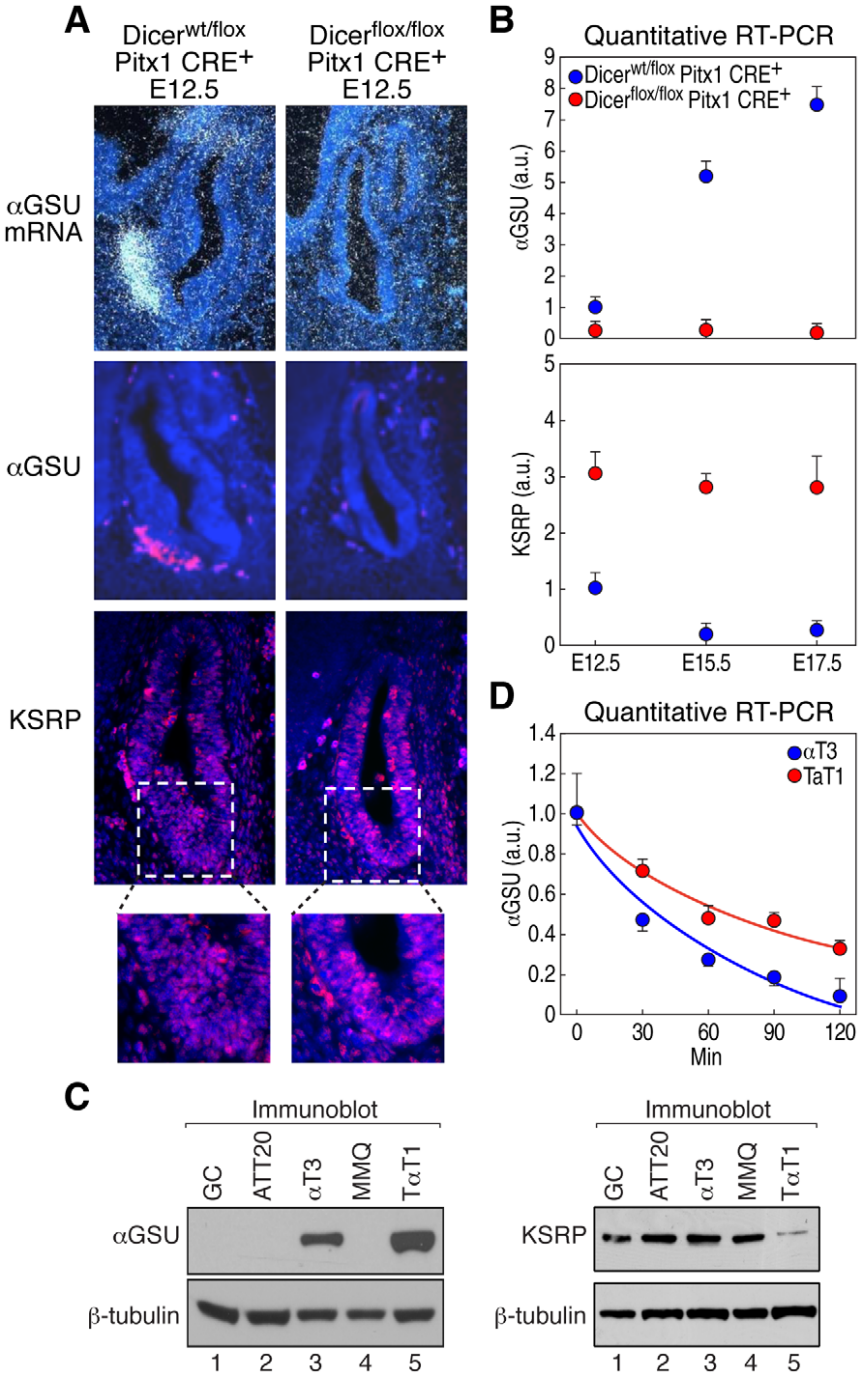
Dicer regulates αGSU and KSRP expression during pituitary development. (A) *In situ* hybridization (upper panels) and immunohistochemical analyses of αGSU (middle panels) and KSRP (lower panels) in control or *Dicer*-deleted pituitaries at E12.5; a representative sagittal section of pituitary gland is shown. (B) Reverse transcription (RT) followed by quantitative PCR to analyze αGSU (upper panel) and KSRP (lower panel) mRNA expression in control or *Dicer*-deleted pituitaries at E12.5, E15.5, and E17.5. The data were normalized by β2-MG mRNA. a.u., arbitrary units compared to the value of Dicer^wt/flox^ at E12.5. (C) immunoblot analysis of αGSU, KSRP and β-tubulin from several pituitary cell lines as indicated (GC-somatotrope, ATT20-corticotrope, αT3-1-gonadotrope, MMQ-lactotrope, and TαT1-thyrotrope). (D) Quantitative RT-PCR analysis of αGSU transcript in αT3-1 and TαT-1 cells. Total RNA was isolated at the indicated times after addition of actinomycin D. The data were normalized by β2-MG mRNA. All data are presented as mean and s.d. (n = 4).

αGSU expression, which is expressed in pituitary from E12.5 [21] was also undetectable at early embryonic stages of *Dicer*-deleted pituitaries (Figure 3A, 3B). αGSU expression is known to be differentially regulated in gonadotropes and thyrotropes during development [22], while mice harboring a knockout of αGSU display hypertrophy and hyperplasia of the anterior pituitary [23]. In addition, deregulation of αGSU expression was observed in different pituitary adenomas [24], suggesting that αGSU expression is finely regulated in development and pathological conditions. Because the expression of transcription factors that control αGSU promoter activity, such as Gata binding protein 2 (Gata2), Pituitary homeobox 1 and 2 (Pitx1/2), LIM-homeodomain transcription factors (Lhx3 and Lhx4), and Upstream stimulatory factor 1 (USF1) [14], were either unchanged or slightly decreased in *Dicer*-deleted pituitaries at E12.5 (Figure S2A, S2B and data not shown) we tested the hypothesis that αGSU may be post-transcriptionally regulated. In favor of this hypothesis, αGSU mRNA is a labile transcript that contains a canonical ARE (AUUUA) in its 3′UTR (Figure S2C) and displays a half-life that increases during pituitary development [25]. Interestingly, mRNA profiling analysis and quantitative RT-PCR from microdissected pituitaries during development revealed that KSRP mRNA was significantly upregulated in *Dicer*-deleted pituitaries compared to control (Figure 3B and Table S1), indicating that KSRP expression is also under regulatory control of Dicer during pituitary development. KSRP, in turn, may control the decay rate of αGSU mRNA. Indeed, KSRP expression displayed an inverse temporal relationship to αGSU mRNA expression during pituitary development. In particular, between E12.5 and E15.5, KSRP mRNA levels significantly declined while αGSU mRNA level increased nearly fivefold (Figure 3B). In contrast, between E15.5 and E17.5 KSRP mRNA levels did not change while αGSU mRNA level slightly increased nearly 1.4 fold, which may indicate a transcriptional control of αGSU expression and/or an accumulation of the stabilized αGSU transcript in the later phases of pituitary development. Moreover, KSRP expression, evaluated by immunofluorescence, is upregulated in *Dicer*-deleted pituitaries, including in the αGSU expression area (Figure 3A). We then utilized two pituitary-derived cell lines expressing endogenous αGSU, the gonadotrope αT3-1 cell line and the thyrotrope TαT-1 cell line (Figure 3C and Figure S2D) to measure the expression levels of αGSU and KSRP. Strikingly, αT3-1 cells express significantly higher levels of KSRP than T αT1 cells (Figure 3C), consistent with the lower expression and the shorter half-life of endogenous αGSU mRNA in αT3-1 cells (Figure 3C, 3D and Figure S2D). Indeed, quantitative RT-PCR analyses demonstrated that αGSU mRNA was more unstable in αT3-1 than in T αT-1 cells displaying a half-life upon actinomycin D treatment of approximately 30 and 75 minutes (min), respectively (Figure 3D). Therefore, this data suggest that KSRP regulates αGSU expression in pituitary development.

**Figure 4:**
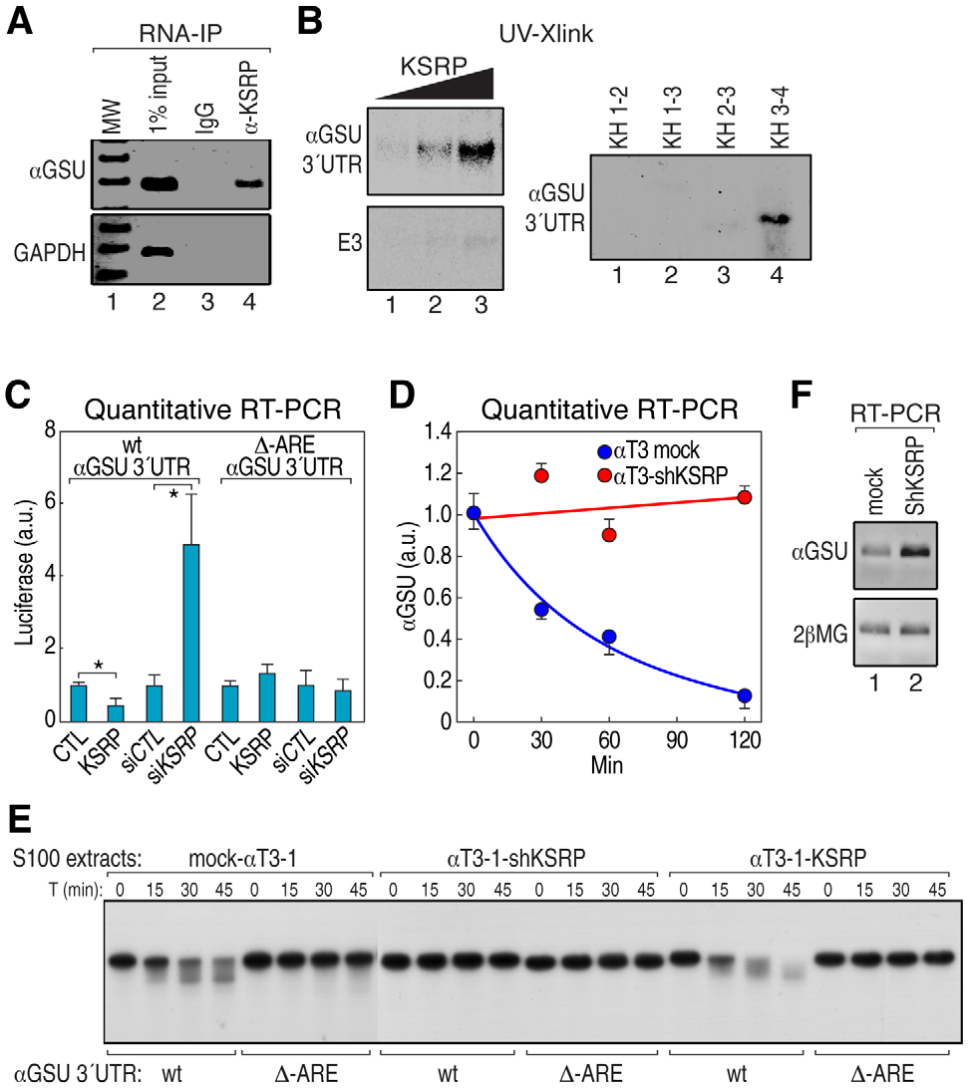
KSRP is required for αGSU mRNA degradation. (A) Anti-KSRP antibody immunoprecipitates αGSU mRNA in αT3-1 cell extracts. GAPDH, glyceraldehyde-3-phosphate dehydrogenase. (B) UV-crosslink assay to analyze the interaction of the recombinant KSRP (40–200 nM) as well as the indicated KSRP deletion mutants (150 nM) with radiolabeled αGSU 3′UTR or pitx2 3′UTR stable region (E3) as a negative control [38]. (C) Luciferase reporter plasmid bearing either wt or Δ-ARE αGSU 3′UTR sequences were cotransfected into HeLa cells with either a vector overexpressing KSRP or siRNA knocking it down. Quantitative RT-PCR analysis of luciferase transcript was normalized using Renilla mRNA. siCTL, control siRNA (D) Quantitative RT-PCR analysis of αGSU transcript in either sh-KSRP or empty pSUPER vector stably transfected into αT3-1 cells. Total RNA was isolated at the indicated times after addition of actinomycin D. The data were normalized by β2-MG mRNA. (E) *In vitro* RNA degradation assays were performed by incubating S100s from mock αT3-1, αT3-1–shKSRP, or -overexpressing KSRP cells with either wt or Δ-ARE mutant αGSU 3′UTR internally ^32^P-labeled and capped RNA substrates. The decay was monitored at the indicated times. (F) RT-PCR analysis of both αGSU and β2-MG transcripts from either mock αT3-1 or αT3-1–shKSRP cells. Student's t-test: *P<0.05. All data are presented as mean and s.d. (n = 4).

To assess whether αGSU transcript is directly regulated by KSRP, we first performed an RNA immunoprecipitation experiment (RIP) from αT3-1 cell extract and found that anti-KSRP antibody immunoprecipitated αGSU mRNA but not the control GAPDH mRNA (Figure 4A). We then demonstrated that recombinant KSRP directly interacted with αGSU 3′UTR in a concentration-dependent fashion and that, as expected [26], KH3–4 domains accounted for the highest affinity binding to αGSU 3′UTR (Figure 4B and Figure S3A). To determine whether KSRP controls αGSU mRNA half-life, we constructed reporter plasmids in which αGSU 3′UTR was cloned downstream a luciferase open reading frame (ORF) with or without a mutation that deletes the ARE sequence motif. Then, we cotransfected the luciferase reporter with either a vector overexpressing KSRP or a siRNA to silence it (si-KSRP) into either human HeLa or mouse NIH-3T3 cells (Figure S3B). KSRP significantly promoted the downregulation of both luciferase mRNA and activity of the construct containing wt αGSU 3′UTR, but not the mutant, while cotransfection with either empty plasmid or siRNA control had no effect (Figure 4C and Figure S3C, S3D). We then investigated whether KSRP controls the half-life of endogenous αGSU mRNA in stably transfected KSRP knockdown αT3-1 cells (αT3-1–shKSRP) [27]. Indeed, αGSU mRNA was more stable in αT3-1–shKSRP compared to the mock-transfected cells (Figure 4D). *In vitro* degradation experiments using αT3-1 cell extracts from mock, overexpressing KSRP or sh-KSRP stable cells demonstrated that ARE is essential for KSRP-dependent control of αGSU mRNA decay. As presented in Figure 4E, KSRP promoted rapid decay rate of αGSU 3′UTR, but not the mutant substrate. Finally, KSRP knockdown in αT3-1 cells led to a more than sevenfold increase of the steady-state level of αGSU mRNA as compared to control cells (Figure 4F). These data indicate that in pituitary development Dicer modulates the turnover rate of αGSU mRNA through regulation of KSRP expression, resulting in a significant increase of the steady-state level of αGSU mRNA-protein.

**Figure 5:**
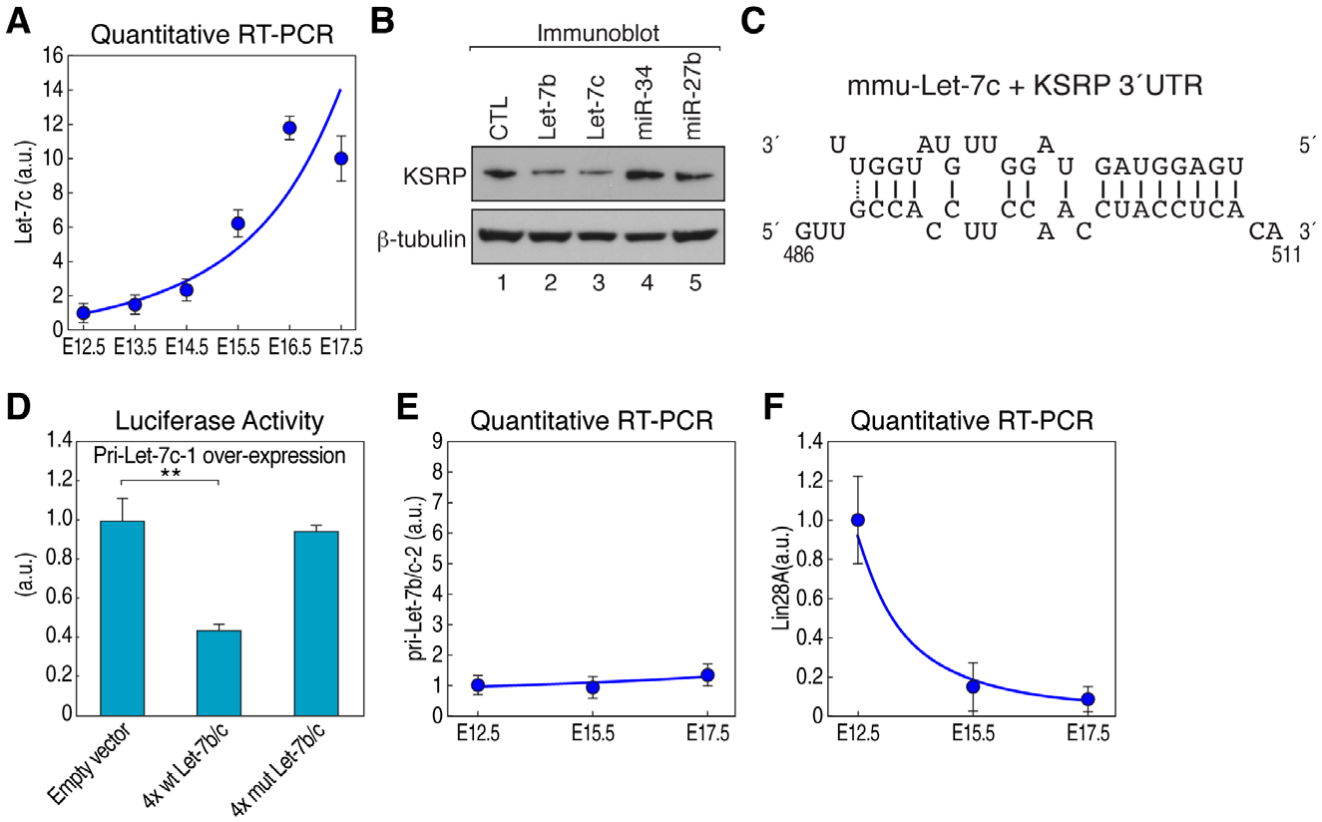
Let-7b and let-7c directly downregulate KSRP mRNA during pituitary development. (A) Ontogeny of let-7c expression in pituitary development by quantitative RT-PCR. The data were normalized by U6 RNA. (B) Immunoblot analysis of KSRP and β-tubulin in NIH-3T3 cells singularly transfected with the indicated miRNA mimics. (C) RNA duplex expected to result from base pairing of KSRP mRNA with let-7c. (D) Relative luciferase activity of reporter constructs containing four either wt or mutant let-7b/c binding sites from KSRP 3′UTR sequence in 293T cells overexpressing pri-let-7c-1. The data were normalized using Renilla activity. (E) Ontogeny of pri-let-7b/c-2, and (F) Lin28A mRNA expression in pituitary development by quantitative RT-PCR. The data were normalized by β2-MG mRNA. Student's t-test: **P<0.01. All data are presented as mean and s.d. (n = 4).

In order to identify candidate miRNAs that may directly regulate KSRP mRNA during pituitary development, we used three different algorithms, namely Target Scan (http://www.targetscan.org/), miRanda (http://cbio.mskcc.org/mirnaviewer/), and PicTar (http://pictar.mdc-berlin.de/). Eight candidate miRNAs targeting KSRP 3′UTR were predicted by these programs (Figure S4A), but only let-7b, let-7c, miR-24, miR-27a, and miR-27b were expressed during pituitary development, as evaluated by a miRNA profiling array experiment (Table S2). Strikingly, quantitative RT-PCR data demonstrated that only let-7b/c expression display an inverse temporal relationship to KSRP mRNA during pituitary development (Figure 3B, Figure 5A, and Figure S4B, S4C, S4D). Furthermore, to determine which miRNAs can suppress endogenous KSRP mRNA expression, NIH-3T3 cells were singularly transfected with let-7b, let-7c, miR-24 or miR-27b mimic and the steady-state KSRP expression level was assayed by western blot and quantitative RT-PCR. KSRP expression levels were significantly decreased in presence of let-7b/c (Figure 5B and Figure S4E), while the expression of other ARE-BPs, including TTP and HuR, was not affected (Figure S4F, S4G). In addition, let7b/c knockdown significantly increased KSRP protein levels in NIH-3T3 cells (Figure S4H), overall indicating that let-7b/c may directly regulate KSRP expression. According to bioinformatic predictions, there is a single let-7b/c binding site in KSRP 3′UTR (Figure 5C and Figure S4I). To assess whether KSRP mRNA is directly targeted by let-7b/c, we constructed reporter plasmids in which KSRP 3′UTR fragments were cloned downstream to a luciferase ORF (Figure S5A). Overexpression of either pri-let-7b or pri-let-7c-1 significantly reduced the luciferase activity in 293T cells transfected with the construct containing KSRP 3′UTR segment A, which possesses the predicted let-7b/c binding site (Figure S5A–S5D). In addition, both pri-let-7b and pri-let-7c-1 reduced the expression of the luciferase mRNA encoded by the construct containing KSRP 3′UTR segment A (Figure S5E). Importantly, both pri-let-7b and pri-let-7c-1 overexpression in 293T cells significantly repressed luciferase activity in presence of a reporter construct containing the wild-type (wt) multimerized binding site for let-7b/c, but not the mutant reporter construct (Figure 5D, Figure S5F, Table S3). *In vitro* degradation experiments using cell extracts from NIH-3T3 cells transfected with let-7b/c mimics and controls confirmed that let-7b/c induce KSRP mRNA degradation (Figure S5G). These data suggest that let-7b/c directly regulate KSRP mRNA expression.

**Figure 6:**
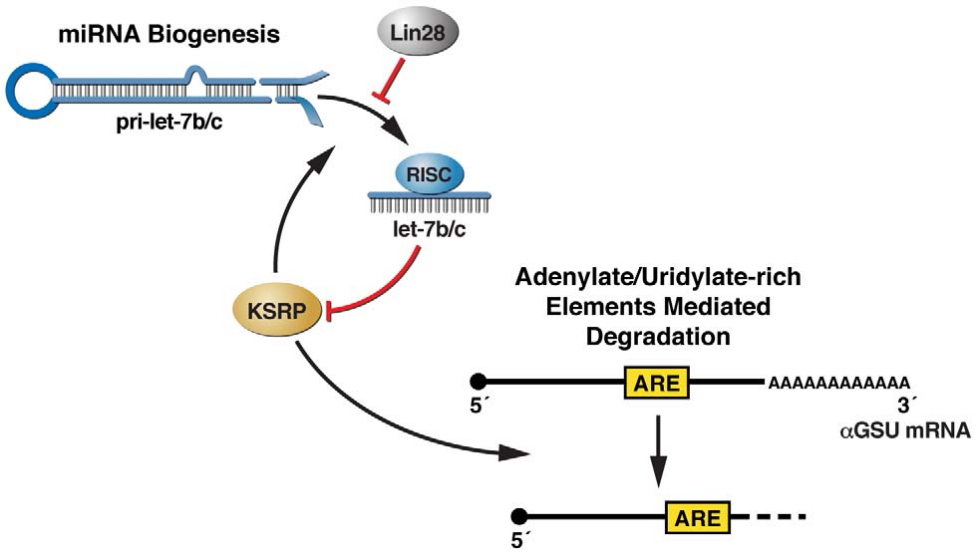
A model for the miRNA–dependent control of αGSU transcript stability during pituitary development.

Let-7c originates from two different transcripts, namely pri-let-7c-1 and pri-let-7c-2, while let-7b originates from a unique transcript, pri-let-7b, which forms a cluster with pri-let-7c-2. Interestingly, while mature let-7b/c was significantly upregulated during pituitary development, pri-let-7-c1 and pri-let-7b/c2 levels showed a poor increase, suggesting that a post-transcriptional control of let-7b/c expression occurs during pituitary development (Figure 5E and Figure S6). In accord with this hypothesis, we found that the expression of the mRNA encoding for Lin28A, which blocks let-7 biogenesis [28], [29], has an inverse temporal relationship to let-7b/c expression (Figure 5A, 5F and Figure S4B). This observation suggests that Lin28A may postranscriptionally regulate let-7 expression during pituitary development. Importantly, we and others have recently reported that, besides its activity promoting mRNA decay, KSRP is able to favor miRNA maturation [12], [30], [31], [32]. Among miRNA precursors the processing of which is regulated by KSRP, we found the let-7 family [30], [32]. KSRP recognizes an evolutionary conserved “G” triplet in the terminal loop sequence of let7 precursors to promote the processing. Therefore, we propose a model in which let-7b/c operate within a negative feedback loop in pituitary development. In concert with the reduction of Lin28A, KSRP specifically induces the biogenesis of let-7b/c, and, in turn, KSRP expression is post-transcriptionally downregulated by let-7b/c. This downregulation of KSRP expression ultimately affects the decay of KSRP-target labile mRNAs, as we demonstrated for αGSU mRNA (Figure 6). In conclusion, these data reveal the existence of an unsuspected KSRP-dependent molecular strategy for αGSU mRNA regulation during pituitary development, promoting on the one hand the biogenesis of microRNAs and on the other hand the degradation of unstable mRNAs, based on its versatile ability to recognize different classes of RNA motifs [26].

miRNA- and AMD-based gene silencing mechanisms have emerged as two key post-transcriptional pathways that function on a genome-wide scale to modulate tissue development and differentiation [1], [5]. The interplay between these two pathways has been demonstrated in cell culture models in which changes in AMD activity occur upon downregulation of HuR that is directly controlled by cell-specific miRNAs [6], [8]. Some ARE-BPs, including HuR and TTP, may contribute to the degree of miRNA-targeting complexity by associating with RISC and either promoting [9], [33] or inhibiting [7] the silencing of target mRNAs. Moreover, some miRNAs, including miR-369-3p and miR-466l, directly target ARE and compete with TTP for ARE binding to prevent mRNA degradation mediated by AMD [34], [35], uncovering additional layers of complexity for biochemical crosstalk between the two pathways. Here we identified in an animal model a post-transcriptional regulatory network operative during pituitary development in which mRNA stability of the pituitary hormone αGSU is significantly increased through let7b/c-mediated regulation of KSRP expression. Interestingly, some other ARE-BPs, including TTP and HuR, examined also demonstrated a Dicer-dependent expression regulation in pituitary development, indicating a large potential for linked programs of post-transcriptional regulation (Figure S7). Altogether, these findings define a unanticipated functional crosstalk between miRNA- and AMD- dependent gene regulation during mammalian organogenesis events.

## Materials and Methods

### Mice

Animals were kept in a pathogen-free barrier facility and maintained in accordance with Institutional Animal Care and Use Protocol of University of California at San Diego (UCSD) in accordance with appropriate national regulations concerning animal welfare. *Dicer^flox/flox^, Pitx1 CRE^+^* mutant embryos were obtained by crossing *Dicer^flox/flox^* mice [16] with mice heterozygous for floxed *Dicer* and *Pitx1 CRE^+^* [17]. *Dicer* knockout animals were genotyped by PCR using primers indicated in Table S3.

### *In situ* hybridization and immunohistochemistry

*In situ* hybridization and immunofluorescence were carried out as previously described [36]. Mouse embryos from E12.5 to E17.5 were fixed in 10% neutral formalin, penetrated with 20% sucrose in PBS, and embedded in OCT compound. Serial 12-µm sections were hybridized with ^35^S-labeled antisense RNA probes. The probes used in this study were generated by RT-PCR from various tissues and verified by sequencing. For immunofluorescence staining, the sections were boiled for 10 min in 10 mM citrate buffer (pH 6.0) to retrieve antigens and stained with mouse Ki-67 (Pharmingen, 1∶50), BrdU (ICN Biomedicals, 1∶20), KSRP (1∶50), rabbit polyclonal antibodies against cleaved caspase-3 (Cell Signaling, 1∶200), GH (DAKO, 1∶200), TSHβ (National Hormone and Pituitary Program, National Institute of Diabetes and Digestive and Kidney Diseases, rabbit, 1∶400), ACTH (Sigma, 1∶100), αGSU (Novus Biologicals, 1∶200), and LHβ (1∶200). Secondary Alexa Fluor 488-, and Alexa Fluor 594-conjugated antibodies were from Jackson ImmunoResearch and Molecular Probes. Slides were coverslipped in Vectashield Mounting Medium with DAPI (Vector Laboratories). The results were analyzed on a Zeiss Axioplan2 microscope with a Hamamatsu camera.

### Microarray analysis

For mRNA profiling analysis pituitaries were microdissected from 8 specimens of either *Dicer* knockout or wt at E12.5, the total RNA was isolated by using RNAeasy kit (Qiagen) and the Agilent whole genome array platform was used. The normalized data were analyzed with Biometric Research Branch (BRB) array tools 3.8.0 [37]. Signal intensity below 500 was discarded. Scatterplots of log2-transformed signal intensities were used to identify probes with at least 1.2 fold change.

MicroRNA profiling analysis was provided by LC Sciences (Houston, TX, USA). Total RNAs from 30 microdissected pituitaries at E12.5 and E17.5 were isolated using miRNAeasy kit (Qiagen) to interrogate a human/mouse/rat miRNA array (LC Sciences) comprising a total of 1256 unique mature miRNAs (837 human, 599 mouse, and 350 rat, based on Sanger miRBase 11.0; Sanger Institute, Hinxton, UK). Data were analyzed by first subtracting the background and then normalizing the signals using a LOWESS filter (locally weighted regression).

### Cell culture and transfections

αT3-1, NIH-3T3, HeLa, MMQ, GC, TαT-1, ATT20, and HEK293T (293T) cells were cultured in DMEM containing 4500 mg/L glucose, 110 mg/L pyruvate, and 548 mg/L L-glutamine (Gibco) supplemented with 10% fetal bovine serum (Gibco), and 1% pen/strep antibiotics (Gibco). Cells were transiently transfected for 48 hours with Lipofectamine 2000 (Invitrogen) according to the manufacturer's instructions. miRNA mimics for control, let-7b, let-7c, miR-24, and miR-27b (Qiagene) as well as miRNA inhibitors for control, let-7b, let-7c, miR-24, and miR-27b (Qiagene) were singularly transfected at 100 nM final concentration. siRNA scramble sequence control, siRNAs against human and mouse KSRP were transfected at the final concentration of 24 nM.

Additional methods are provided in Text S1.

## Supporting Information

Figure S1 Dicer promotes cell survival during pituitary development. (A) RT-PCR analysis of Dicer mRNA in control or *Dicer*-deleted pituitaries at E12.5. (B) Northern blotting for let-7c and U6 from control or *Dicer*-deleted pituitaries at E17.5. (C,D) Immunohistochemical analysis of c-Casp-3 and Ki67 in control or *Dicer*-deleted pituitaries at E12.5; a representative sagittal section of pituitary gland is shown. The star indicates a non-specific immunostaining.
(TIF)

Figure S2 Dicer regulates transcriptional regulatory programmes in pituitary development. (A) Expression of USF1, Lhx3, Lhx4 and Pitx1 mRNAs in control or *Dicer*-deleted pituitaries at E12.5 by quantitative RT-PCR. The data were normalized by β2-MG mRNA. (B) Expression of Gata2 and Pitx2 in control or *Dicer*-deleted pituitaries at E12.5 and E13.5 by *in situ* hybridization; a representative sagittal section of pituitary gland is shown. (C) αGSU 3′UTR with the AUUUA pentamer ARE sequence motif in red. (D) RT-PCR analysis of αGSU and β2-MG (beta 2-microglobulin, control transcript) expression from several pituitary cell lines as indicated (GC-somatotrope, ATT20-corticotrope, αT3-1-gonadotrope, MMQ-lactotrope, and TαT1-thyrotrope). Student's t-test: *P<0.05. All data are presented as mean and s.d. (n = 4).
(TIF)

Figure S3 KSRP promotes the degradation of αGSU mRNA. (A) The UV-crosslinking reactions were subjected to immunoblot with either anti-KSRP (upper panel) or anti-GST (lower panel). (B) Immunoblot analysis of total extracts from either HeLa (upper-left) or NIH-3T3 (upper-right) cells transiently transfected with either scramble siRNA (siCtrl), or human or mouse KSRP siRNA (si-KSRP). In the lower panel HeLa cells were transfected with either pcDNA-3 or a pcDNA-3 overexpressing HA-KSRP. (C) KSRP knockdown in HeLa cells reduced the luciferase activity of a reporter construct bearing αGSU 3′UTR sequence. Control, transfection of pcDNA-3. The data were normalized using Renilla activity. (D) Quantitative RT-PCR analysis of luciferase transcript bearing either wt or Δ-ARE αGSU 3′UTR cotransfected into NIH-3T3 cells with either KSRP siRNA or control. The data were normalized using Renilla mRNA. Student's t-test: *P<0.05, **P<0.01. All data are presented as mean and s.d. (n = 4).
(TIF)

Figure S4 Let-7b/c control KSRP expression. (A) Schematic representation of the KSRP 3′UTR with the bioinformatically predicted targeting miRNAs. (B) Ontogeny of mature let-7b, (C) miR-27b, and (D) miR-24 expression during pituitary development by quantitative RT-PCR. The data were normalized by U6 RNA. (E) Quantitative RT-PCR analysis of KSRP, (F) TTP or (G) HuR mRNAs in NIH-3T3 cells transfected with the indicated miRNA mimics. The data were normalized by β2-MG mRNA. (H) Immunoblot analysis of KSRP and β-tubulin in NIH-3T3 cells singularly transfected with the indicated miRNA inhibitors. (I) RNA duplex expected to result from base pairing of KSRP mRNA with let-7b. All data are presented as mean and s.d. (n = 4).
(TIF)

Figure S5 KSRP mRNA is directly regulated by let-7b/c. (A) Relative luciferase activity of reporter constructs containing KSRP 3′UTR segments in 293T cells overexpressing pri-let-7c-1. Schematic representation of the KSRP 3′UTR and the location of A, B, and C is shown at the bottom. The data were normalized using Renilla activity. (B) Northern blot analysis of let-7c and let-7b in 293T cells overexpressing either pri-let-7c-1 or pri-let-7b. (C,D) Relative luciferase activity of reporter constructs bearing the indicated segments of KSRP 3′UTR sequences in 293T cells cotransfected with either pri-let-7b or an empty pcDNA-3 vector as mock. The data were normalized using Renilla activity. (E) Luciferase reporter plasmid bearing KSRP 3′UTR A was cotransfected into 293T cells with either pri-let-7c-1 or pri-let-7b. Quantitative RT-PCR analysis of luciferase transcript was normalized using Renilla mRNA. (F) Relative luciferase activity of reporter constructs containing four wt or mutant let-7b/c binding sites from KSRP 3′UTR sequence in 293T cells overexpressing pri-let-7b. The data were normalized using Renilla activity. (G) *In vitro* RNA degradation assays were performed by incubating S100s from let-7b/c overexpressing-NIH-3T3 cells and control cells with four wt or mutant let-7b/c binding sites from KSRP 3′UTR sequence internally ^32^P-labeled and capped RNA substrates. The decay was monitored at the indicated times. Student's t-test: *P<0.05; **P<0.01. All data are presented as mean and s.d. (n = 4).
(TIF)

Figure S6 Ontogeny of pri-let-7c-1 expression in pituitary development. Reverse transcription (RT) followed by quantitative PCR to analyze by quantitative RT-PCR to analyze pri-let-7c-1 expression in pituitary at E12.5, E15.5 and E17.5. The data were normalized by β2-MG mRNA. All data are presented as mean and s.d. (n = 4).
(TIF)

Figure S7 Dicer-dependent expression profile of different ARE-BPs during pituitary development. Reverse transcription (RT) followed by quantitative PCR to analyze TTP, HuR, TIA-1, and AUF1 mRNA expression in control or *Dicer*-deleted pituitaries at E12.5, E15.5 and E17.5. The data were normalized by β2-MG mRNA. a.u., arbitrary units compared to the value of Dicer^wt/flox^ at E12.5. All data are presented as mean and s.d. (n = 4).
(TIF)

Table S1 mRNA profiling microarray data from *Dicer*-deleted microdissected pituiries and control at E12.5. mRNAs whose expression levels changed of at least 1.2 fold in *Dicer*-deleted pituitaries compared to control at E12.5 are listed.
(XLS)

Table S2 miRNAs profiling analysis in pituitaries at E12.5 and E17.5.
(XLS)

Table S3 Mouse Oligonucleotides used for RT-PCR, cloning and Northern blot analysis of U6 RNA.
(DOC)

Text S1 Supporting Methods
(DOC)
