# Supplementary figures and images for: Let-7b/c Enhance the Stability of a Tissue-Specific mRNA during Mammalian Organogenesis as Part of a Feedback Loop Involving KSRP
Source: PLoS Genet. 2012 Jul 26;8(7):e1002823. doi: 10.1371/journal.pgen.1002823 (PMC3405994; doi:10.1371/journal.pgen.1002823)

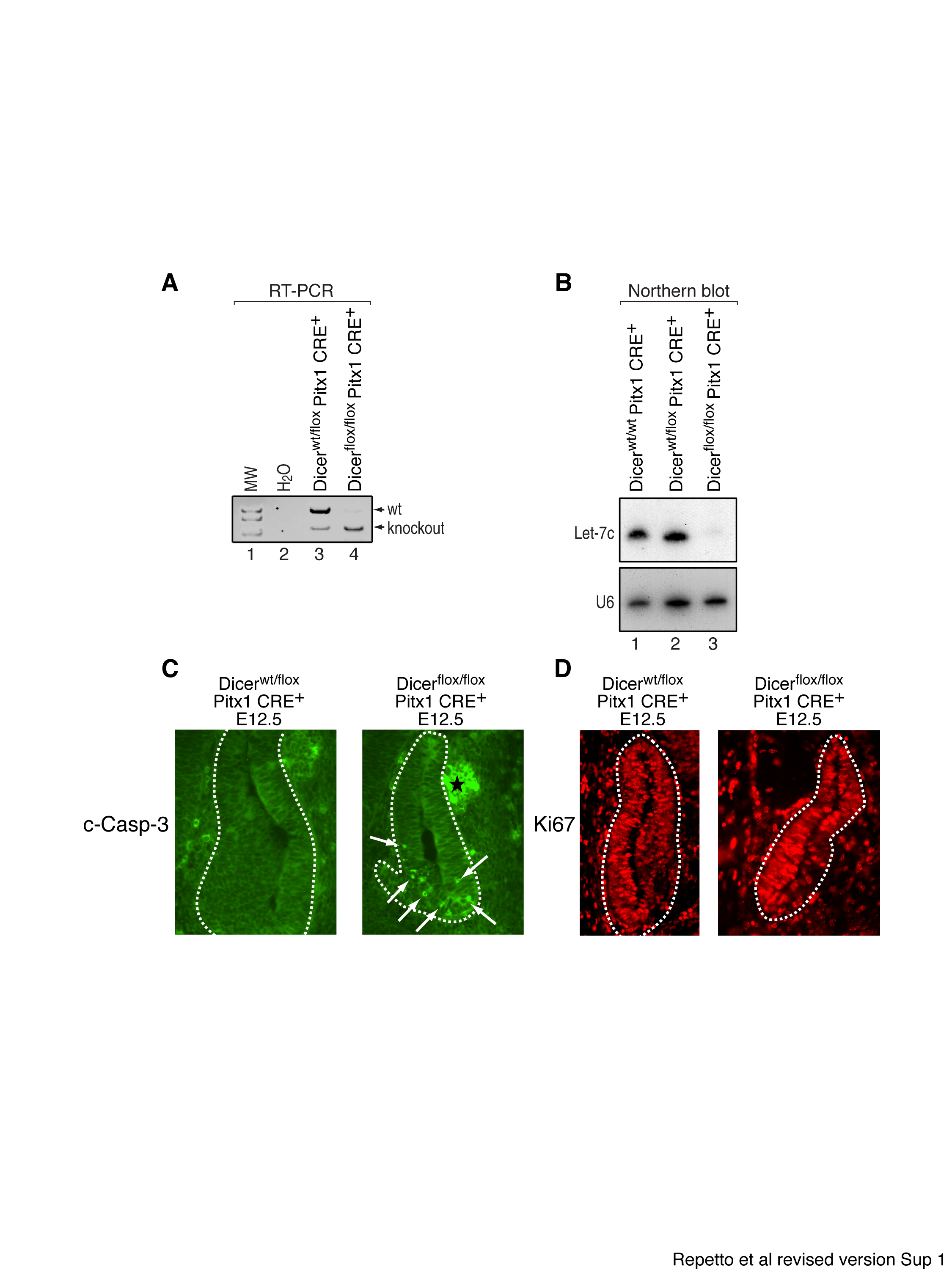

Supplement: Figure S1 — Dicer promotes cell survival during pituitary development. (A) RT-PCR analysis of Dicer mRNA in control or Dicer-deleted pituitaries at E12.5. (B) Northern blotting for let-7c and U6 from control or Dicer-deleted pituitaries at E17.5. (C,D) Immunohistochemical analysis of c-Casp-3 and Ki67 in control or Dicer-deleted pituitaries at E12.5; a representative sagittal section of pituitary gland is shown. The star indicates a non-specific immunostaining. (TIF) [file pgen.1002823.s001.tif]

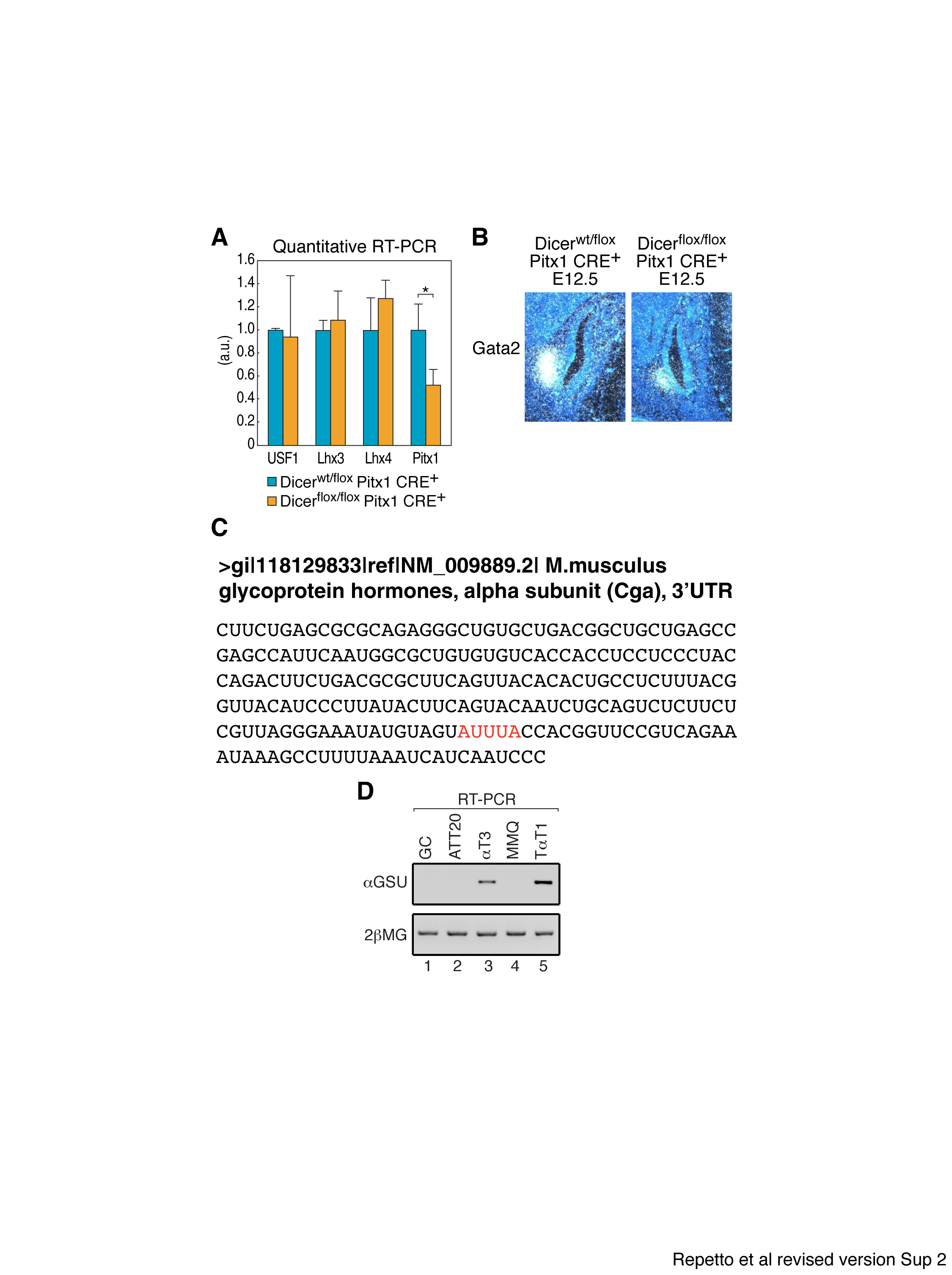

Supplement: Figure S2 — Dicer regulates transcriptional regulatory programmes in pituitary development. (A) Expression of USF1, Lhx3, Lhx4 and Pitx1 mRNAs in control or Dicer-deleted pituitaries at E12.5 by quantitative RT-PCR. The data were normalized by β2-MG mRNA. (B) Expression of Gata2 and Pitx2 in control or Dicer-deleted pituitaries at E12.5 and E13.5 by in situ hybridization; a representative sagittal section of pituitary gland is shown. (C) αGSU 3′UTR with the AUUUA pentamer ARE sequence motif in red. (D) RT-PCR analysis of αGSU and β2-MG (beta 2-microglobulin, control transcript) expression from several pituitary cell lines as indicated (GC-somatotrope, ATT20-corticotrope, αT3-1-gonadotrope, MMQ-lactotrope, and TαT1-thyrotrope). Student's t-test: *P<0.05. All data are presented as mean and s.d. (n = 4). (TIF) [file pgen.1002823.s002.tif]

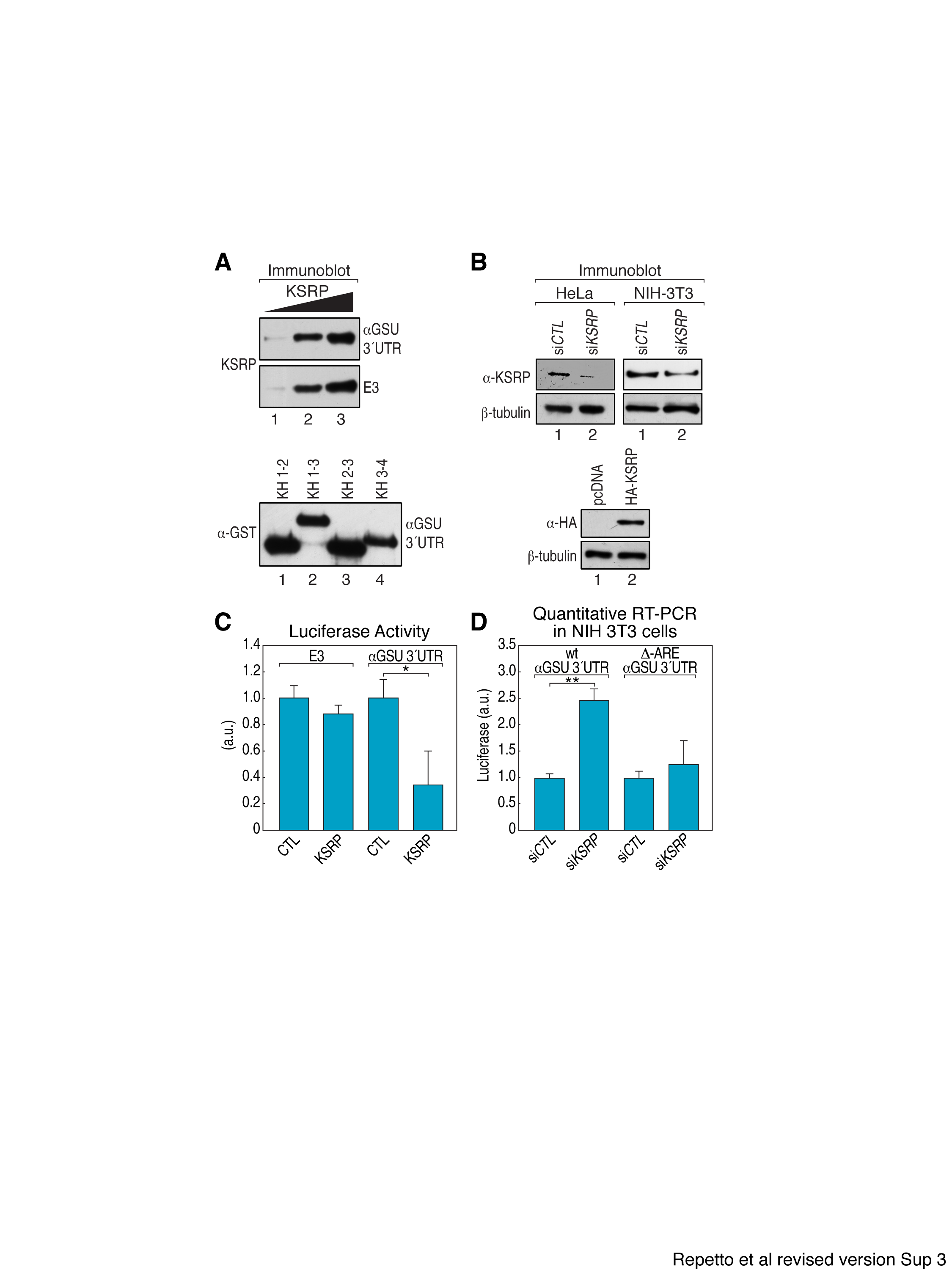

Supplement: Figure S3 — KSRP promotes the degradation of αGSU mRNA. (A) The UV-crosslinking reactions were subjected to immunoblot with either anti-KSRP (upper panel) or anti-GST (lower panel). (B) Immunoblot analysis of total extracts from either HeLa (upper-left) or NIH-3T3 (upper-right) cells transiently transfected with either scramble siRNA (siCtrl), or human or mouse KSRP siRNA (si-KSRP). In the lower panel HeLa cells were transfected with either pcDNA-3 or a pcDNA-3 overexpressing HA-KSRP. (C) KSRP knockdown in HeLa cells reduced the luciferase activity of a reporter construct bearing αGSU 3′UTR sequence. Control, transfection of pcDNA-3. The data were normalized using Renilla activity. (D) Quantitative RT-PCR analysis of luciferase transcript bearing either wt or Δ-ARE αGSU 3′UTR cotransfected into NIH-3T3 cells with either KSRP siRNA or control. The data were normalized using Renilla mRNA. Student's t-test: *P<0.05, **P<0.01. All data are presented as mean and s.d. (n = 4). (TIF) [file pgen.1002823.s003.tif]

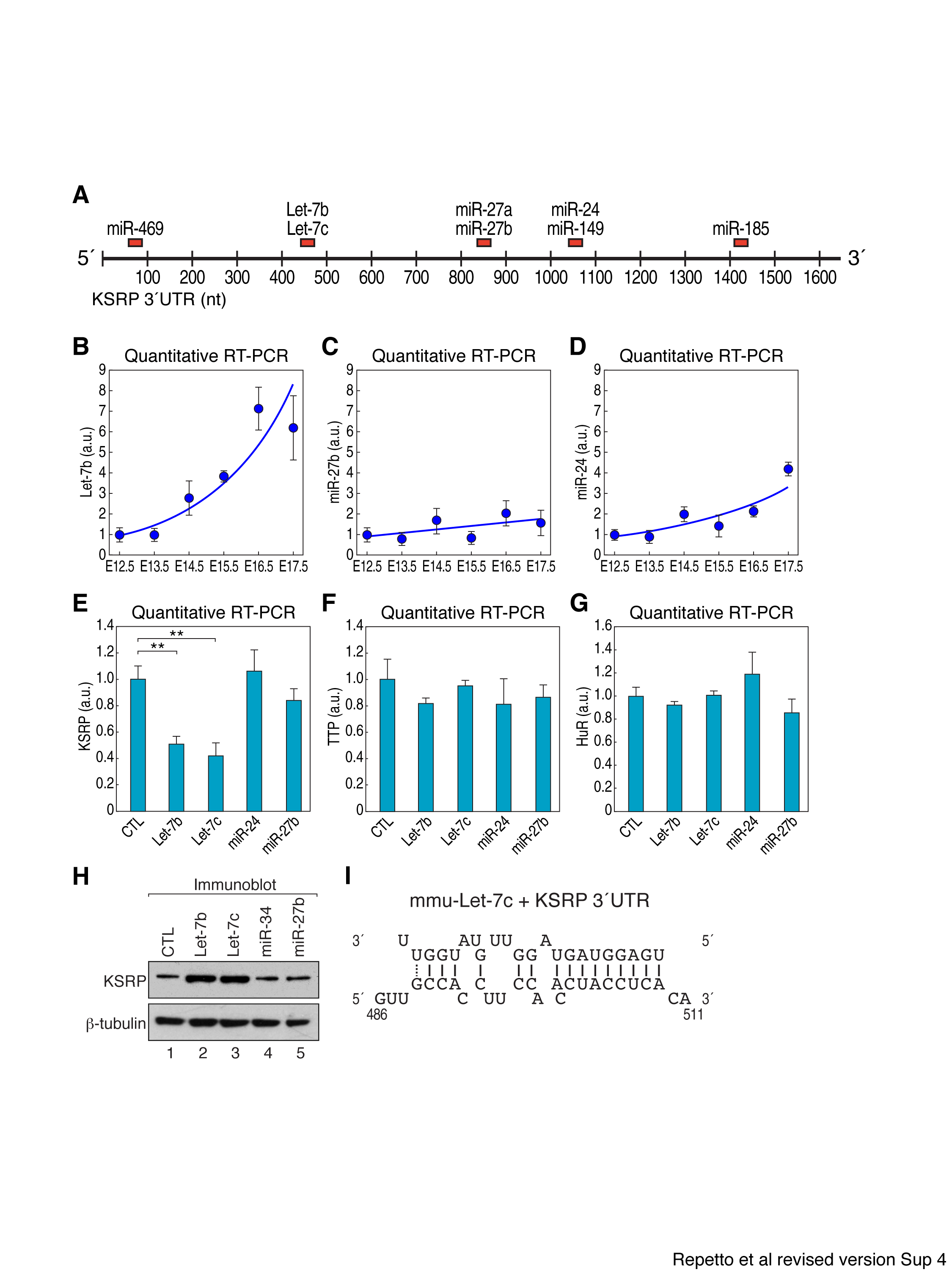

Supplement: Figure S4 — Let-7b/c control KSRP expression. (A) Schematic representation of the KSRP 3′UTR with the bioinformatically predicted targeting miRNAs. (B) Ontogeny of mature let-7b, (C) miR-27b, and (D) miR-24 expression during pituitary development by quantitative RT-PCR. The data were normalized by U6 RNA. (E) Quantitative RT-PCR analysis of KSRP, (F) TTP or (G) HuR mRNAs in NIH-3T3 cells transfected with the indicated miRNA mimics. The data were normalized by β2-MG mRNA. (H) Immunoblot analysis of KSRP and β-tubulin in NIH-3T3 cells singularly transfected with the indicated miRNA inhibitors. (I) RNA duplex expected to result from base pairing of KSRP mRNA with let-7b. All data are presented as mean and s.d. (n = 4). (TIF) [file pgen.1002823.s004.tif]

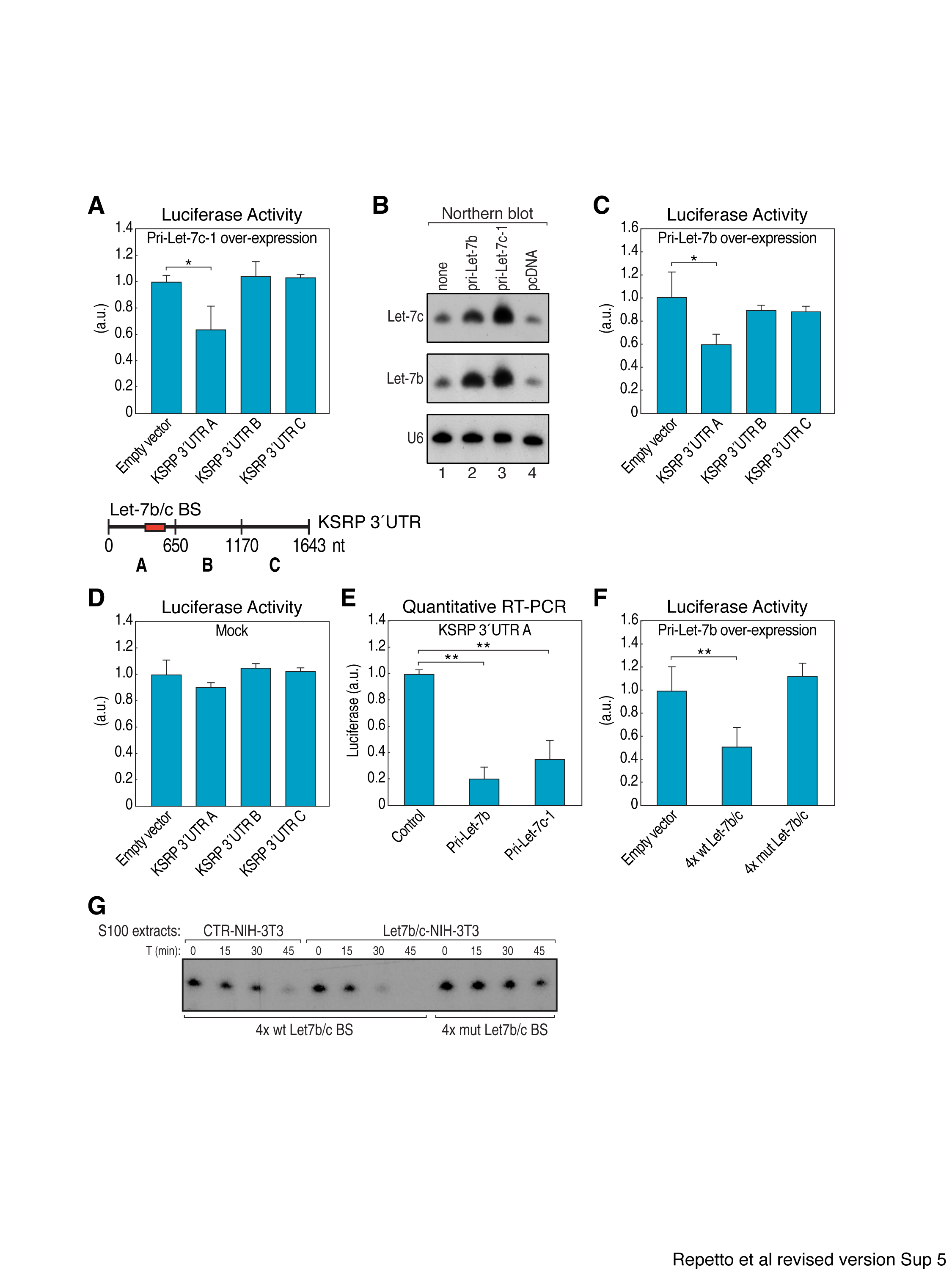

Supplement: Figure S5 — KSRP mRNA is directly regulated by let-7b/c. (A) Relative luciferase activity of reporter constructs containing KSRP 3′UTR segments in 293T cells overexpressing pri-let-7c-1. Schematic representation of the KSRP 3′UTR and the location of A, B, and C is shown at the bottom. The data were normalized using Renilla activity. (B) Northern blot analysis of let-7c and let-7b in 293T cells overexpressing either pri-let-7c-1 or pri-let-7b. (C,D) Relative luciferase activity of reporter constructs bearing the indicated segments of KSRP 3′UTR sequences in 293T cells cotransfected with either pri-let-7b or an empty pcDNA-3 vector as mock. The data were normalized using Renilla activity. (E) Luciferase reporter plasmid bearing KSRP 3′UTR A was cotransfected into 293T cells with either pri-let-7c-1 or pri-let-7b. Quantitative RT-PCR analysis of luciferase transcript was normalized using Renilla mRNA. (F) Relative luciferase activity of reporter constructs containing four wt or mutant let-7b/c binding sites from KSRP 3′UTR sequence in 293T cells overexpressing pri-let-7b. The data were normalized using Renilla activity. (G) In vitro RNA degradation assays were performed by incubating S100s from let-7b/c overexpressing-NIH-3T3 cells and control cells with four wt or mutant let-7b/c binding sites from KSRP 3′UTR sequence internally 32P-labeled and capped RNA substrates. The decay was monitored at the indicated times. Student's t-test: *P<0.05; **P<0.01. All data are presented as mean and s.d. (n = 4). (TIF) [file pgen.1002823.s005.tif]

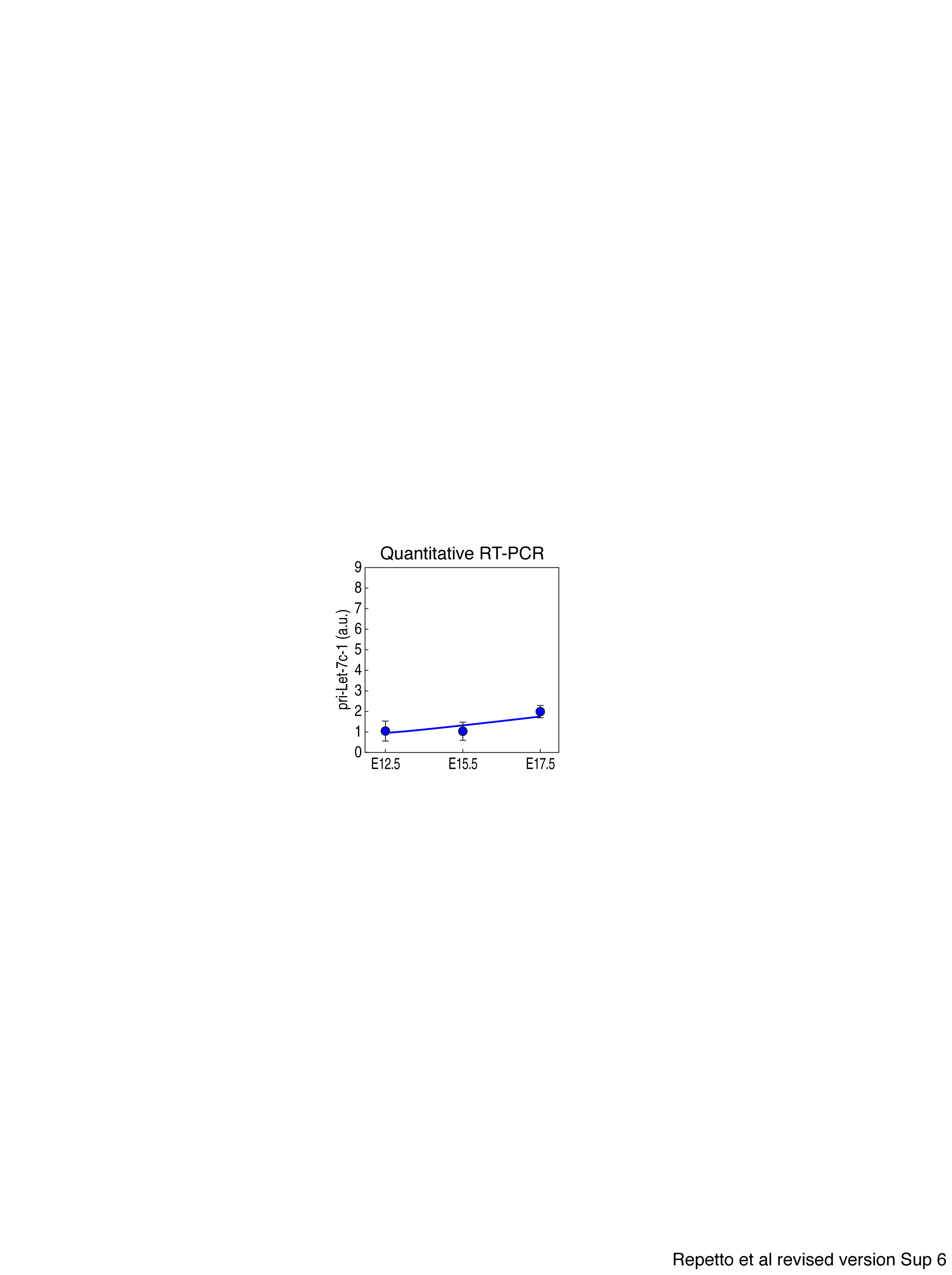

Supplement: Figure S6 — Ontogeny of pri-let-7c-1 expression in pituitary development. Reverse transcription (RT) followed by quantitative PCR to analyze by quantitative RT-PCR to analyze pri-let-7c-1 expression in pituitary at E12.5, E15.5 and E17.5. The data were normalized by β2-MG mRNA. All data are presented as mean and s.d. (n = 4). (TIF) [file pgen.1002823.s006.tif]

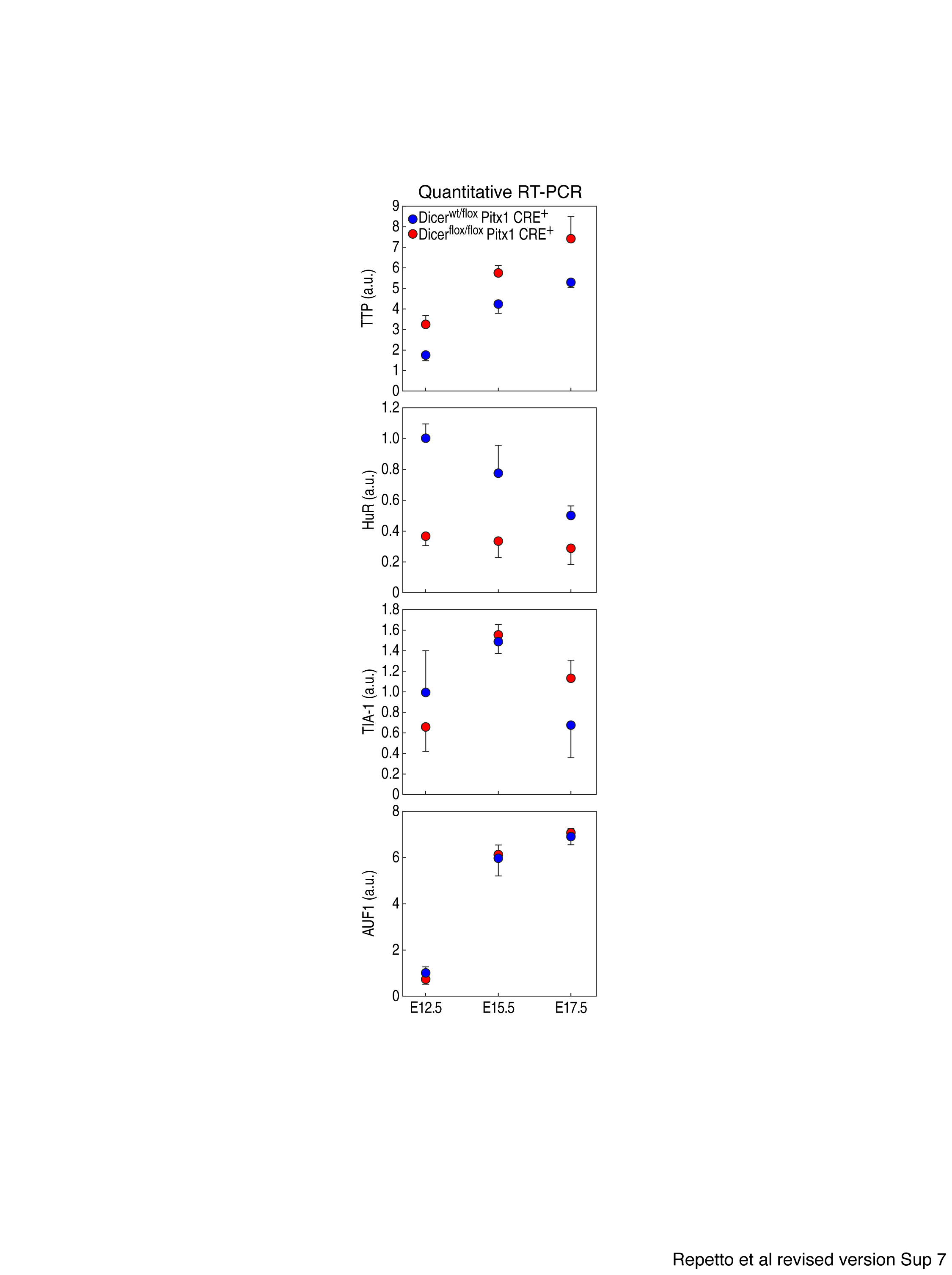

Supplement: Figure S7 — Dicer-dependent expression profile of different ARE-BPs during pituitary development. Reverse transcription (RT) followed by quantitative PCR to analyze TTP, HuR, TIA-1, and AUF1 mRNA expression in control or Dicer-deleted pituitaries at E12.5, E15.5 and E17.5. The data were normalized by β2-MG mRNA. a.u., arbitrary units compared to the value of Dicerwt/flox at E12.5. All data are presented as mean and s.d. (n = 4). (TIF) [file pgen.1002823.s007.tif]
