# Supplementary material for: Let-7b/c Enhance the Stability of a Tissue-Specific mRNA during Mammalian Organogenesis as Part of a Feedback Loop Involving KSRP
Source: PLoS Genet. 2012 Jul 26;8(7):e1002823. doi: 10.1371/journal.pgen.1002823 (PMC3405994; doi:10.1371/journal.pgen.1002823)
Supplement: Table S3 — Mouse Oligonucleotides used for RT-PCR, cloning and Northern blot analysis of U6 RNA. (DOC) [file pgen.1002823.s010.doc]

Table S3

|  | Forward primer | Reverse primer |
| --- | --- | --- |
| αGSU (RT-PCR) | 5’-TCATGCTGTCCATGTTCCTGC-3’ | 5’-cactctggcatttcccattactg-3’ |
| Dicer (genotyping) | 5’-CCTGACAGTGACGGTCCAAAG-3’ | 5’-CATGACTCTTCAACTCAAACT-3’ |
| CRE (genotyping) | 5’-ATCGCCAGGCGTTTTCTGAGCATA-3’ | 5’-ggatccgccgcataaccagtga-3’ |
| Dicer (RT-PCR) | 5’-AACAAAAGAGCCTTTCAGGGA-3’ | 5’-TTAGGAACCTGAGGCTGGTTA-3’ |
| 2βMG | 5’-AGTTAAGCATGCCAGTATGGCC-3’ | 5’-TTCTTTCTGCGTGCATAAATTGTAT-3’ |
| GAPDH | 5’-AAGCAACATAGACGTTGTCGC-3’ | 5’-AATCAACACCTTCTTCGCACC-3’ |
| αGSU (qPCR) | 5’-GTGTATGGGCTGTTGCTTC-3’ | 5’-CATGTGGCCTCCGAGGTAA-3’ |
| pri-let-7c-1 (qPCR & cloning) | 5’-ATTCTATCTACAACCTTGCCA-3’ | 5’-AACAGCCCGTGAGAAATAG-3’ |
| pri-let-7b (qPCR & cloning) | 5’-TATGGCTCTAGTGGCCTGT-3’ | 5’- TTTATTTATACCCAGGTCCCA-3’ |
| Lin28A (qPCR) | 5’-GAGTTCACCTTTAAGAAGTCT-3’ | 5’-GTTGTAGCACCTGTCTCCT-3’ |
| KSRP (qPCR) | 5’-CTTTCAACCCTGGACCCTT-3’ | 5’-GCATTAGGATCTGTAGCCG-3’ |
| TTP (q-PCR) | 5’-GCCACAAGTTCTACCTCCA-3’ | 5’-GAGAAGCTGATGCTTTGTC-3’ |
| HuR (q-PCR) | 5’-TACACCACCAGGCACAGAG-3’ | 5’-AAGGTTGTAGATGAAGATGC-3’ |
| TIA-1 (q-PCR) | 5’-CAGTGGCTTGGTGGAAGAC-3’ | 5’-GACTAGACTGACTCACAACC-3’ |
| AUF1 (q-PCR) | 5’-GGAGAGTGTAGATAAGGTCAT-3’ | 5’-GGCTCTTTTGTTTTCATGGC-3’ |
| luciferase (q-PCR) | 5’-CGTCGCCAGTCAAGTAACAACC-3’ | 5’-CACGGCGATCTTTCCGCC-3’ |
| renilla (q-PCR) | 5’-TGCAGAAGTTGGTCGTGAGGCA -3’ | 5’-TCTAGCCTTAAGAGCTGTAATTGAACTGG-3’ |
| USF (q-PCR) | 5’-ACAAGATCAACAACTGGATTG-3’ | 5’-CAGCTGCAACTGATCTAAC-3’ |
| Lhx3 (q-PCR) | 5’-GAGTTCTACCTCATGGAAGAC-3’ | 5’-CTTGGGCGAAGTGTTGTAG-3’ |
| Lhx4 (q-PCR) | 5’-GGCGGACAGTTAATGAATG-3’ | 5’-TGATGCCCAGATTACTGTCC-3’ |
| Pitx1 (q-PCR) | 5’-CTCCTACAACAACTGGGCG-3’ | 5’-AGGTTGTTGATGTTGTTGAGG-3’ |
| αGSU 3’UTR (cloning) | 5’-CTTCTGAGCGCGCAGAGGGCTG-3’ | 5’-GGGATTGATGATTTAAAAGG-3’ |
| KSRP 3’UTR A (cloning) | 5’-GATGGGTGCAGAGGCTTAC-3’ | 5’-AAGAGGAGGAGAAGTCTGC-3’ |
| KSRP 3’UTR B (cloning) | 5’-CCTCTTCCTCTTCCTCCTG-3’ | 5’-TGTTCTGCTCTTTGTGATTT-3’ |
| KSRP 3’UTR C (cloning) | 5’-TAAATCACAAAGAGCAGAACA-3’ | 5’-TGATACCATTTATTTGTGAAG-3’ |
| wt Let-7c BS from KSRP 3’UTR (cloning) | 5’-CGATCGTTGCCACCTTCCAACCTACCTCACAGGGGT-3’ |  |
| mutant Let-7c BS from KSRP 3’UTR (cloning) | 5’-CGATCGTTGCCACCTTCCAACgatTTagtCAGGGGT-3’ |  |
| U6 |  | 5’-CGTTCCAATTTTAGTATATGTGCTGCCGAAGCGAGCAC-3’ |
