# Supplementary material for: Let-7b/c Enhance the Stability of a Tissue-Specific mRNA during Mammalian Organogenesis as Part of a Feedback Loop Involving KSRP
Source: PLoS Genet. 2012 Jul 26;8(7):e1002823. doi: 10.1371/journal.pgen.1002823 (PMC3405994; doi:10.1371/journal.pgen.1002823)
Supplement: Text S1 — Supporting Methods (DOC) [file pgen.1002823.s011.doc]

**Supporting Methods**

TUNEL assay

TUNEL assay was performed using In Situ Cell Death Detection Kit (Roche) according to the manufacturer’s instructions.

BrdU incorporation

2 mg of Bromodeoxyuridine (BrdU; Sigma) diluted in sterile 1X PBS at 10 mg/ml concentration was intraperitoneally injected in pregnant mice 24 hrs prior to sacrifice the animals.

Luciferase reporter assay

293T, HeLa or NIH-3T3 cells (80% confluence in 12-well plates) were transfected with Lipofectamine 2000 according to the manufacturer’s instructions. Luciferase reporter assay was performed as previously described [32].

RNA in Vitro Degradation and UV-Crosslinking

^32^P-labeled RNAs were synthesized and used as substrates for either *in vitro* degradation assays or UV-crosslinking experiments as previously described by Chen et al. [39].

RNA immunoprecipitation

RNA immunoprecipitation was performed as previously described [32]. Briefly, cells lysates were immunoprecipitated with Protein A-Sepharose-coupled antibodies at 4ºC overnight. Total RNA was prepared using Trizol (Invitrogen), retrotranscribed using random primers and amplified by PCR. The primer sequences are detailed in Table S3.

Northern blot

Total RNA was isolated from cells using Trizol, resolved on 10% polyacrylamide-urea gels, and electroblotted onto HyBond N+ membranes. Membranes were hybridized overnight with radiolabeled DNA oligonucleotide antisense to either let-7c or let-7b in ExpressHyb solution (Clontech). After hybridization, membranes were washed three times with 2X SSC and 0.05% SDS, twice with 0.1X SSC and 0.1% SDS, exposed overnight to imaging screens, and analyzed using a Storm 860 PhosphorImager. The same blot was hybridized (upon stripping in boiling 0.1% SDS) with three distinct probes, including control U6 RNA (Table S3).

Protein, mRNA, and miRNA expression analysis

Protein expression by western blot analysis and RNA expression by semiquantitative and quantitative RT-PCR was carried out by using standard procedures. Briefly, for mRNA semiquantitative and quantitative RT-PCR analysis, total RNA was isolated from either 240 microdissected pituitaries at different time embryonic stages (each day from E12.5 to E17.5) or cells using RNeasy kit (Qiagen) and cDNA was synthesized with an oligo-dT using Superscript III (Invitrogen). For miRNA quantitative RT-PCR analysis, small RNA was isolated from microdissected pituitaries using Purelink kit (Invitrogen) and cDNA was synthesized using N-code kit (Invitrogen). Quantitative RT-PCR using Sybr Green was performed on a Mx3000P QPCR system (Stratagen). The primer sequences are detailed in Table S3.

Recombinant proteins and antibodies for western blot

Production of recombinant KSRP and its deletion mutants have been previously described [39]. Affinity-purified rabbit polyclonal anti-KSRP antibody was previously described [40]. Mouse monoclonal anti-GST was purchased from Chemicon. Anti-β-tubulin and anti-HA mouse monoclonal were purchased from Sigma.

siRNAs

In order to knock down KSRP, these siRNAs were synthesized by Qiagen:

- human KSRP 5'- GAUCAACCGGAGAGCAAGAUU -3',
- mouse KSRP 5’-GGACAGUUUCACGACAACG-3’

**Supporting references**

39. Chen CY, Gherzi R, Ong SE, Chan EL, Raijmakers R, et al. (2001) AU binding proteins recruit the exosome to degrade ARE-containing mRNAs. Cell 107: 451-464.

40. Gherzi R, Lee KY, Briata P, Wegmuller D, Moroni C, et al. (2004) A KH domain RNA binding protein, KSRP, promotes ARE-directed mRNA turnover by recruiting the degradation machinery. Mol Cell 14: 571-583.
